# Genome-Wide Association Studies of Hypertension: Light at the End of the Tunnel

**DOI:** 10.4061/2010/509581

**Published:** 2010-04-29

**Authors:** Claire E. Hastie, Sandosh Padmanabhan, Anna F. Dominiczak

**Affiliations:** British Heart Foundation Cardiovascular Research Centre, University of Glasgow, 126 University Place, Glasgow G12 8TA, UK

## Abstract

Despite its significant genetic component, the study of hypertension by genome-wide association presents more challenges than other common complex diseases. Its high prevalence, heterogeneity, and somewhat unclear definition are the challenges that need to be overcome on one hand. On the other hand, there are issues of small effect sizes and pleiotropism that are not specific to hypertension alone but nonetheless magnify the problems of genetic dissection when coupled with phenotypic misclassification. We discuss issues of study design and summarise published genome-wide association studies (GWASs) of hypertension and blood pressure. With careful study design and analysis success is possible, as demonstrated by the recent large-scale studies. Following these, there is still further scope to advance the field through high fidelity phenotyping and deep sequencing.

## 1. Introduction

Hypertension is the major factor responsible for the most deaths worldwide (around 7 million or 12.8% per year) and this is 46% more than tobacco usage, the next major risk factor [1]. From an epidemiological and clinical perspective, blood pressure at the higher end of the normal population distribution is associated with an increased risk of cardiovascular mortality and morbidity. But clinical risk assessment is based on a predefined threshold at which the quantitative blood pressure phenotype in converted into a binary trait (hypertension) and management strategies are directed towards blood pressure reduction below this threshold at which the risk of excess cardiovascular events is abolished. Hence large-scale efforts to dissect the genetic underpinnings of hypertension are justified. While the causation of hypertension is multifactorial with both genetic and environmental components, the purpose of this paper is to discuss the genetic determinants of high blood pressure and GWAS study design. 

 For any genetic study, the key initial step is phenotypic specification. Here whether studying blood pressure as a quantitative trait or the qualitative hypertension phenotype depends on whether one follows the Pickering argument that blood pressure is inherited as a “graded character,” and hence a complex non-Mendelian trait, or the Platt suggestion that hypertension is a qualitative abnormality and a simple Mendelian disease [2]. The observed normal unimodal distribution of blood pressure and its complex multifactorial aetiology support studies of blood pressure as a quantitative phenotype. According to the latter argument, if hypertension is a dichotomous risk trait, one would expect a bimodal distribution and this is not observed. However both approaches are useful and complementary in the genetic dissection of this trait which has so far been extremely resistant to the GWAS approach.

## 2. Evidence for a Genetic Component in Hypertension

There are multiple strands of evidence showing that genetic factors contribute to blood pressure and hypertension. Firstly, the normal distribution of blood pressure in the general population indicates the presence of multiple environmental and genetic factors and thus a polygenic aetiology. Secondly, rare monogenic forms of hypertension associated with major defects in renal salt handling prove that gene mutations can cause hypertension leading to a hypothesis that minor variations in these genes may contribute to the common essential hypertension. Finally from a population perspective, there is considerable evidence from twins and family aggregation studies indicating the presence of a heritable component.

 It is estimated that around 30% of variation in blood pressure is due to genetic factors [3]. Hypertension is about twice as common in individuals who have one or two hypertensive parents, and blood pressure is more closely correlated in monozygotic than dizygotic twins [4, 5]. In the Montreal Adoption Study investigators compared blood pressure correlation between biological sibling pairs and adoptive sibling pairs (as well as parent-child correlations). SBP correlation coefficients were 0.38 and 0.16 for biological and adopted siblings, respectively, and DBP coefficients 0.53 compared with 0.29 respectively [6]. 

 Two measures that are commonly used to assess the genetic component of a trait are heritability (*h^2^*) which is the fraction of variation in disease susceptibility due to genetic factors, and sibling recurrent risk (*λ* *s*) which is the degree of elevated risk of disease for a sibling of an affected individual compared with a member of the general population. The heritability of clinic systolic blood pressure is around 15–40% and 15–30% for clinic diastolic blood pressure [7, 8]; whereas for ambulatory night-time systolic and diastolic BP the heritabilities are 69% and 51% [7]. It is pertinent to point out that though the heritability estimates are considerable, this does not equate to magnitude of genetic effect. This is because the denominator in the estimate of heritability comprises measurement error and variances attributable to genes, shared environment, unshared environment and unmeasured determinants. This is illustrated by the example above where minimizing measurement errors by using ambulatory night-time values inflates the heritability estimates. Heritability is also a property of the population studied and low heritability estimates would suggest that genetic mapping would be difficult for that phenotype. The sibling recurrent risk of hypertension is around 1.2–1.5 [9], indicating a phenotype with modest genetic effect.

## 3. Strategies for Gene Mapping of Complex Diseases

The field of common complex disease genetics has in recent years moved from linkage to association study design because association analysis has far greater power to detect variants of modest effect and of lower frequency. The initial successes of hypertension gene mapping have come from studies of monogenic syndromes. The monogenic diseases are examples of diseases which are under very strong negative selection primarily because they affect fitness and are less likely to be transmitted to the next generation. The monogenic Mendelian forms of hypertension are thus rare, and responsible gene mutations are highly penetrant, and under very strong selection which keeps them at low frequencies with high levels of allelic heterogeneity. Thus these are highly amenable to linkage analysis. In contrast, susceptibility variants involved in essential hypertension are likely to have low or medium penetrance and are probably not subject to such strong selection resulting in lower allelic heterogeneity and greater prevalence of the trait. Thus linkage analysis as expected has not really provided robust validated loci for hypertension. To detect loci conferring a genotypic relative risk of 1.5 (minor allele frequency (MAF) = 0.1) by linkage analysis requires an estimated 67,816 affected sibling pairs (ASPs) whereas detection is possible through association with just 2218 singletons [10]. Moreover it is easier to recruit participants from the general population (than families as required for linkage), and there are fewer sampling restrictions in some disease categories such as late onset. 

 Association studies are typically performed in unrelated population samples (although it is possible to conduct them on related individuals). For qualitative traits they measure statistical association between a disease (phenotype) and genetic marker (genotype) directly by comparing allele frequencies of cases and controls. The aim is to establish whether a particular allele occurs in cases, compared with controls, more frequently than expected by chance. Quantitative traits, for example, blood pressure, cholesterol, and glucose, are assessed for association using linear regression. GWAS is typically an indirect or map-based approach. This measures the association between a phenotype and a marker allele (or “tag” SNP), which is correlated with the true causal allele due to linkage disequilibrium (LD). Linkage disequilibrium is defined as “the statistical association, within gametes in a population, of the alleles at two loci” [11] (on the same chromosome). It is assumed that typically a causal variant will not have been typed in GWAS which take advantage of LD to genotype a set of tag SNPs as proxies for the entire set of SNPs. The amount of LD between two loci is summarised by the metric *r* ^2^ (squared correlation coefficient for each SNP) which varies between 0 and 1. The maximum *r* ^2^ can be used to translate coverage (measure of how well the SNPs that are part of a genotyping set capture all known variants) to the sample size that is required for an indirect association study. To cover unobserved loci well an *r* ^2^ value of ≥0.8 with typed loci is considered sufficient [12]. In general SNPs in LD are more likely to be inherited together because they are physically close to each other on the chromosome. But this is not necessarily the case; studies have shown that levels of local LD vary, with some adjacent SNPs being independent despite their proximity and others of ≥100 kb apart being in a high or a significant LD [13]. Patterns of LD are affected by many factors such as population growth, population structure, admixture, natural selection, genetic drift, rate of recombination and mutation, and gene conversion [14]. 

 Traditionally association studies tested hypotheses based on candidate genes, for which there was prior evidence (of known physiological pathways that affect the phenotype in question) that a genetic variant influenced disease risk. Unsurprisingly many of the candidate genes for hypertension are involved in sodium balance. No candidate gene study has yet demonstrated a reproducible association with hypertension [15]. There are several potential reasons for this, which highlight the drawbacks of candidate gene studies:

- the wrong genes may have been selected;
- the causative genes may be upstream or downstream from the genes studied;
- discovery of genetic variants in novel pathways is not possible as candidate gene studies rely on a priori information regarding disease mechanisms.

In addition to the above there are the possibilities of population stratification, phenotypic and locus heterogeneity, and insufficient sample size: problems common to candidate gene studies and GWAS. Finally the SNPs studied might not provide complete coverage of the variants within the genes.

## 4. The GWAS Strategy

The chance of detecting genetic variants that influence common disease depends on the underlying genetic architecture. That is to say, the number of susceptibility alleles, whether they are common or rare, their frequency, and whether their action is neutral or deleterious. Allelic spectra vary greatly between disease genes. Common alleles and those of high frequency are of course easier to detect, as are deleterious alleles. The entire GWAS strategy is based on the Common Disease Common Variant (CDCV) hypothesis, that the causative genes for common diseases have relatively simple allelic spectra, that is, one or a few predisposing alleles of relatively high frequency. For GWAS it has been suggested that, as a rough guide, SNPs should meet a threshold of MAF ≥ 1% [16] or MAF ≥ 5% [11] to be considered common. As yet there is insufficient empirical evidence to determine the validity of the CDCV hypothesis, and arguments for and against have been put forward. These are crucial to research using SNP mapping to predict common disease risk, which assumes that the theory is by and large accurate (linkage studies of families or ASPs, by contrast, are robust to allelic heterogeneity). 

 A key part of the argument against the CDCV hypothesis rests on the fact that the risk of common disease depends on the interaction of many genes and environmental factors. In particular late-onset disorders of high prevalence in modern western society have been heavily influenced by changes in lifestyle factors such as diet and physical activity, and not by common disease-predisposing alleles. The risk conferred by any one factor, whether genetic or environmental, is weak and most cases are not predominantly determined by genetic variance. On the other hand, the relative risks observed in family members (a more rapid than linear decline in risk with increasing distance of relationship) support the conclusion that the majority of risk is due to a modest number of loci with common disease-predisposing alleles. The existing evidence suggests that alleles of both high and low frequency play a part in common disease [17–22].

 Wang et al. have argued against making a distinction between rare and common disease-predisposing alleles [12]. Instead they propose that the allelic spectrum of disease associated variants be considered in the context of all variants in the human genome. In this framework the neutral model is that the allelic spectrum of disease variants and that of all variants are the same. Most susceptibility variants would be rare (MAF < 1%); however SNPs with MAF > 1% would still account for more than 90% of genetic differences between individuals and therefore make a significant contribution to phenotypic variation [23]. This lies somewhere between the two opposing views regarding the CDCV hypothesis. Common diseases vary in their allelic spectra depending on the evolutionary forces exerted upon them; nevertheless it is estimated that each will likely have hundreds of common and rare variants contributing to their familial clustering [12].

 The International HapMap Project is a global consortium mapping all common SNPs in different populations across the world. The availability of SNP maps from HapMap led to a revolution in the dissection of common diseases and traits based on the common disease common variant hypothesis using the GWAS approach. This is a hypothesis-generating approach where no assumptions are made regarding the location or function of the causal variant. The dense genotyping chips that are now available contain hundreds of thousands of tag SNPs and offer increasingly better coverage of the human genome (whether within or outside genes). Adequate coverage requires ≥300 000 SNPs with more needed for African samples due to greater genetic diversity in those populations [11] and thus less LD [24, 25]. Though there is some concern that a set of tag SNPs that were selected in one population may not perform well in another [11, 14], the availability of dense SNP arrays will overcome this. There is also evidence of good tag SNP transference across populations [26, 27]. This is especially true for different populations within the same continent; the greatest disparities are between African and non-African samples. 

 While GWASs scan the genome for association signals without selecting for gene regions, Jorgenson and Witte have argued for a gene-centric approach to GWAS [28]. The reasons they outline are the following: genic variants are more likely to be functionally important than nongenic; variants in many genes are in lower LD than those outside genes so may be difficult to capture through indirect association. By focusing solely on genes and not the whole genome there is potential to increase coverage of genes and decrease the genotyping burden. The genic approach has greater power to detect variants within genes but suffers from a loss of power for nongenic variants. Despite this Jorgenson and Witte demonstrated empirically using HapMap data that it is more efficient in detecting causal variants than the indirect whole-genome approach when related to genotyping burden. Their suggestion of the best overall GWAS approach is to combine indirect genotyping data with gene-based SNPs in high priority regions, or alternatively to use a more stringent LD threshold in genic regions to “over-capture” genic SNPs.

**Figure 1:**
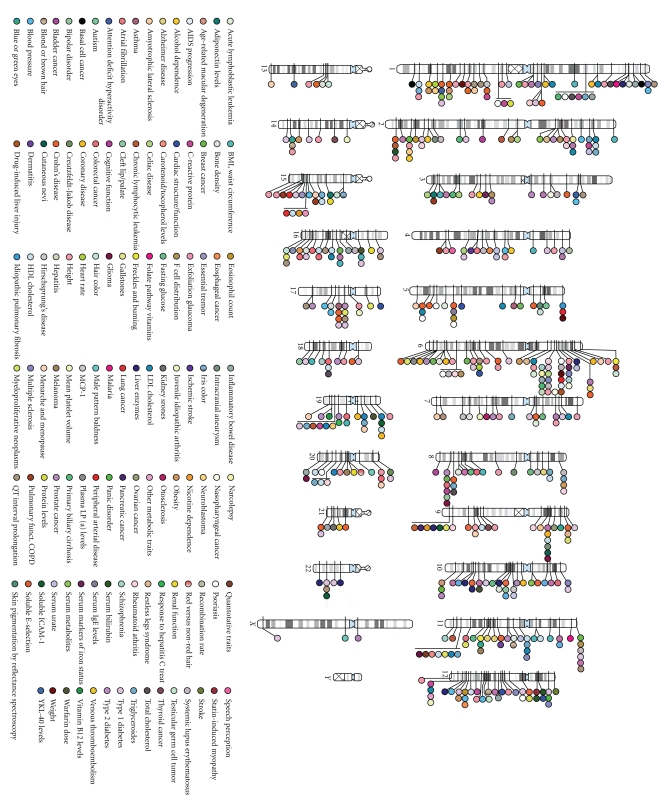
Published genome-wide associations until Sept. 2009. 536 published GWAS at *P* ≤ 5 × 10^−8^ (reproduced with permission from [31]).

 A limitation of GWAS is that they are very expensive, especially with the large sample sizes that are required for small effects. However, technological advancements are rapidly reducing the cost of genotyping. In a bid to further reduce costs, some researchers have adopted a two-stage GWAS study approach. In stage 1 a proportion of the samples are genotyped on all markers, and then in stage 2 a proportion of these markers are genotyped in the remaining samples [29]. Another approach to make studies more economical is the use of common controls for several groups of different disease cases. This was recently demonstrated by the Wellcome Trust Case Control Consortium (WTCCC) [30]. In 2008 Donnelly summarised all GWAS recorded at that time in the National Human Genome Research Institute catalogue [31], which amounted to more than 300 replicated associations for more than 70 common diseases and quantitative traits [32]. As of September 2009, there were more than 500 published GWAS at *P* ≤ 5 × 10^−8^ (see Figure 1).

## 5. Study Design Issues in GWAS

### 5.1. Significance Thresholds

By convention statistical significance using frequentist methods is determined using the *P-*value threshold of .05, with values below this considered significant (i.e., there is evidence to reject the null hypothesis of no effect). This is not appropriate for GWAS because the large number of tests performed increases the chance of type I error. An alternative threshold, proposed by Risch and Merikangas and now widely adopted, is *P* < 5 × 10^−8^, which corresponds to an equivalent false positive rate of 5% for 1 000 000 independent tests of association [10]. This is calculated using the simple Bonferroni correction for multiple testing, which calculates a new significance threshold by dividing .05 by the number of tests performed. In practice this is conservative as it does not take levels of LD into account; the use of tagging SNPs means the genome can be covered sufficiently with around half this number of SNPs (i.e., ~500 000). This threshold is preferable to the traditional .05 but it has been argued that *P *value alone is not adequate for assessing significance. In addition to the possibility of false positives within a study, the issue of multiple testing can be viewed in the context of replication across studies. If several groups publish the same nominally significant association and these are combined, then the problem arises [11].

### 5.2. Statistical Power

Statistical power to detect a phenotype-genotype association is dependent upon the magnitude of effect, the frequency of causal allele(s), and the sample size. Moreover, for indirect association it is not only the disease predisposing allele frequency that matters, but also the marker allele (or tag SNP) frequency, and the power to detect an association is the greatest when these frequencies match [33]. The extent of LD also influences the likelihood of observing an association. However if the effect size is large this is less important with power being high even at low to moderate LD. Effect size for case control studies is measured as an odds ratio (OR), which estimates the odds of an individual in a given exposure group (i.e., with a certain genetic variant) being a case versus being a control. If the OR is significantly greater than 1 then the variant confers susceptibility to the disease, if it is significantly less than 1 then its effect is protective. Unfortunately large effects are usually rare. 

 For common complex diseases most published genetic effects have to date been modest (OR ~ 1.1–1.5) [17, 34], so a reasonable level of LD is necessary as well as the disease allele being common and close to the marker allele frequency. These conditions translate to a feasible sample size of several thousands of cases and controls. An exception, which no doubt increased expectations of similar findings, is the association between APOE4 and late-onset Alzheimer disease [35] for which the allelic OR is 3.3 [33]. As yet only a small percentage of the human genome has been subject to well-designed association study so it is unknown whether the published effect sizes are representative of the genome overall [12]. The effect sizes observed are expected due to the multifactorial nature of the diseases concerned and individually translate to only a small increase in population absolute risk. Multiple common risk variants of small effect have been combined theoretically, however, to construct risk scores of greater practical significance. Studies of the distribution of genetic effect sizes in other species such as rodents, *Drosophila melanogaster*, crops, and livestock suggest that there will be few genetic loci of large effect and many loci of small effect [36–42]. This view is now widely accepted in the field of common disease genetics [43].

### 5.3. Population Stratification

Population stratification acts as a confounder and can result in artefactual evidence of association [44–46]. It occurs when there are two or more strata in a population, and both the risk of disease and the frequency of marker alleles differ between strata. It therefore may appear that the risk of disease is related to the marker alleles when in fact it is not. A similar concept is admixture, which refers to “the mixture of two or more genetically distinct populations” [12]. The International HapMap Project has demonstrated clear genetic differences between geographically separated populations [47]. 

 The effect of stratification on analysis increases with increasing sample size because even modest levels of underlying population structure are amplified [48, 49]. This has particular relevance to GWAS as they are employing larger and larger samples. A final important point is that confounding by population stratification tends to actually decrease (counter intuitively) with increasing number of ethnic groups [50]. This is because the direction of bias may differ between groups so that the overall combined effect is diluted. Whether population stratification is a real concern or not, to avoid any possibility of bias it is now commonplace in studies of unrelated cases and controls to employ stratification detection and correction methods [46]. 

 Aside from matching cases and controls for genetic background and relevant environmental factors as well as possible and straightforward adjustment for ethnic group, a number of possible solutions to population stratification have been proposed. One is genomic control (GC) which employs detection and correction methods, for example, by using a bank of randomly selected markers (preferably > 100 [11]) that are unrelated to the question of interest to assess artefactual association [48, 51–53]. A scaling factor is then applied to the association results to adjust for the level of ethnic variation observed. A similar approach is structure assessment (SA), which too uses unlinked genetic markers for detection but then attempts to match homogeneous subgroups of the sample for association analysis within these subpopulations [54–57]. It is assumed that any significant association observed within a subpopulation cannot be due to population structure; there is an issue, however, about how many subpopulations to apply since they are a theoretical concept [11]. Explicit detection/correction methods by principal components analysis have also been employed [58, 59]. Due to the large number of markers genotyped in GWAS it is possible to detect low levels of stratification. However, a caveat to all of these detection methods is that with a large enough sample size even small biases will be statistically significant and may lead to overcorrection. 

### 5.4. Replication

Many published association findings have failed to be replicated. This is partly due to the so-called “winner's curse,” which is a bias whereby genetic effect size estimates are overestimated in initial discovery studies of disease-predisposing variants. The degree of effect size inflation can be reduced or eliminated by increasing sample size, and several correction methods have been proposed (e.g., [60, 61]). Technical bias can occur if cases and controls are not genotyped and analysed together in the same way. It is also thought that poor choice of controls and population stratification affect data quality and therefore play a part in replication failure. Meta-analyses that have examined replication failure indicate that in the majority of instances false positives are to blame [17, 62]. 

 Replication of GWAS findings is as important as replication of candidate gene associations, if not more so. The large number of SNPs studied in GWAS and resulting volume of statistical tests performed increases the likelihood of observing type I errors, that is, false positives. In 2007 an NCI-NHGRI (National Cancer Institute and the National Human Genome Research Institute in the US) Working Group on Replication in Association Studies published an excellent summary of their recommendations on the reporting of initial association studies and criteria for replication [63]. There are certain situations in which there are insufficient participant numbers for replication, such as rare diseases or environmental exposures. These concerns do not affect most association studies of common diseases, though, so usually replication is advised. A strategy that increases power over that of individual studies and may be more cost effective than replication is meta-analysis of genome wide datasets [64, 65]. This collaborative way of working is increasingly common as investigators attempt to detect loci with smaller effects.

## 6. GWAS of Hypertension/Blood Pressure

To date there have been few GWAS of hypertension and/or blood pressure, with varying degrees of success in detecting associations with genetic variants. The WTCCC, made up of over 50 British research groups, conducted a GWAS study of 2000 cases each for 7 complex diseases of major public health importance; bipolar disorder, coronary artery disease, Crohn's disease, hypertension, rheumatoid arthritis, type 1 diabetes, and type 2 diabetes. These were compared with 3000 shared common controls that came from two sources: 1500 from the 1958 British Birth Cohort and 1500 blood donors that were recruited for the project. The 2000 hypertension cases were unrelated participants from the British Genetics of Hypertension (BRIGHT) study [9]. Over the entire genome there were 21 SNPs identified with *P *values lower than the genome wide significance threshold of 5 × 10^−7^. Of these, 10 were known associations. Unfortunately, of the 7 diseases of interest, hypertension could be described as the loser in that it was not associated with any SNPs at *P* < 5 × 10^−7^. Moreover there was no evidence for any of the variants previously associated with hypertension (at least partly due to some not being well tagged by the Affymetrix chip, for example, promoter of the WNK1 gene). There were, however, a similar number and distribution of marginal results (with *P*-values between 10^−4^ and 10^−7^) to the other case groups. 

 It was speculated that the lack of a positive result for hypertension in the WTCCC study may have been due to poorly tagged variants or that hypertension may have few common risk alleles with larger effect sizes. Furthermore, misclassification bias may have reduced the power of detecting effects. The common controls were not specifically phenotyped for blood pressure. Due to the high prevalence of hypertension and its existence on the continuum of blood pressure some of the controls may have been misclassified cases. It was estimated that the misclassification of 5% of controls (i.e., if 5% of controls were in fact undiagnosed cases) would translate to a loss of power equivalent to a 10% reduction in sample size [30]. This is because of the dilution of any observable genetic difference, caused by the blurring of the distinction between cases and controls. Considering the expense of genome-wide association analysis and the anticipated relatively small effect sizes any reduction in power poses a serious problem. Moreover individuals with blood pressure in the mid-range of normotension (that is not considered to pose a risk clinically) may still be at increased risk in relation to individuals with low-blood pressure. 

 Two recent studies that have had considerably more success are the Global Blood Pressure Genetics (Global BPgen) Consortium study, conducted by Newton-Cheh et al. (2009) [71], and the Cohorts for Heart and Aging Research in Genome Epidemiology (CHARGE) Consortium study, conducted by Levy et al. (2009) [72]. But in contrast to the WTCCC, these were studies of blood pressure as a quantitative trait and on hypertension. Both had very large discovery samples, providing sufficient power to overcome variations in genotyping platform, participant ascertainment and method of blood pressure measurement between component studies. Each dealt with the confounding effect of antihypertensive medication by adding 15 mmHg to recorded SBP and 10 mmHg to DBP for those who were on such treatment. 

 In the Global BPgen discovery GWAS (*N* = 34,433) two SNPs achieved *P* < 5 × 10^−8^ (considered genome wide significance for this analysis). Once these and borderline significant findings were meta-analysed with validation data there were eight loci associated with either DBP or SBP genome-wide. Investigators also assessed whether these loci were associated with hypertension. The initial GWAS was not conducted for hypertension due to lack of power, so instead the significant loci were examined in planned secondary analysis (*N*  range = 57,410–99,802). In the secondary samples all eight alleles showed association with hypertension in the same direction of effect as continuous blood pressure. It should be noted that all of the reported associations translate to a very small change in blood pressure, approximately 1 mmHg per allele SBP or 0.5 mmHg per allele DBP. However, the effects of multiple variants can be combined to produce a meaningful change in population cardiovascular risk.

 Taking a genome wide significance threshold of *P* < 4 × 10^−7^, the CHARGE discovery GWAS (*N* = 29,136) identified 13 significant SNP associations for SBP, 20 for DBP, and ten for hypertension. There is quite a bit of overlap between phenotypes with many of the top hits attaining significance in the same direction of effect for more than one phenotype.

 The top ten loci for SBP, DBP, and hypertension (30 in total) in the CHARGE cohort were checked for significance in the Global BPgen results. One SNP for SBP, four for DBP, and one for hypertension were assessed for independent replication in Global BPgen. Five SNPs out of this six attained *P* < .008, the threshold for external replication in Global BPgen. When the results for the same 30 SNPs in both studies were analysed together by meta-analysis, there were four associations of genome wide significance (*P* < 5 × 10^−8^) for SBP, six for DBP, and one for hypertension. Again effect sizes were very small, approximately 1 mmHg change in SBP per allele or 0.5 mmHg change in DBP per allele. 

 The remaining published studies have demonstrated far less success in identifying genetic variants that are associated with either hypertension, SBP, or DBP. Many have not observed any SNPs that reached genome-wide significance [66, 67, 73–75]. One possible reason for this failure in some studies is that hypertension and/or blood pressure were not the primary trait of interest [73], or not of a priori interest when the cohort was recruited [75] therefore phenotyping may not have been necessarily thorough. Another possibility is that there were not enough SNPs studied to provide sufficient coverage of the whole genome; in some studies fewer than 100 000 SNPs passed quality control measures [66, 67, 74]. 

Table 1 summarises the most significant hits from GWAS of hypertension and/or blood pressure published since 2007 (other than WTCCC, Global BPgen, and CHARGE), along with meta-analysed discovery and replication results if replication was attempted. One study conducted by Sabatti et al. [75] is omitted from the table because the authors did not publish any results for blood pressure, instead reporting that analysis of blood pressure did not produce any genome-wide significant results. However it was not the primary trait of interest; the authors also studied triglycerides, HDL, LDL, CRP, glucose, insulin, and BMI. 

 Levy et al. [66] and Wang et al. [67] failed to find any associations of genome-wide significance but had limited genomic coverage, studying just 70,897 and 79,447 SNPs, respectively. Furthermore the discovery sample employed by Wang et al. was small at 542 participants. Org et al. [68] and Cho et al. [69] also did not find any associations of genome-wide significance.

 The vast majority of GWAS thus far have been conducted on samples of Caucasian individuals of European ancestry. One of the few studies to examine African Americans was that of Adeyemo et al. [70]. Their initial findings were promising; however replication was either not attempted (in the case of SBP) or the replication findings were in the opposite direction of effect (DBP and hypertension).

## 7. Future Directions

Following an initial dearth of positive results from GWAS of hypertension and blood pressure, the methodological advancement and achievements of recent studies provide optimism and guidance for continued work in this area. Where to now in the search for further novel gene polymorphisms? One approach that has been proposed to increase the likelihood of detecting genetic effects is the recruitment of hypercontrols and individuals with severe hypertension [15]. This requires high fidelity phenotyping, with the overall study design comparing cases and controls at the extreme high and low ends, respectively, of the blood pressure distribution. This would markedly reduce the risk of misclassification bias and thus minimise any resultant loss of power. 

 Much of the unexplained variation in blood pressure may be due to rare variants that are undetectable through traditional GWAS case-control study design. However, intensively genotyped samples are becoming available from the 1000 Genomes Project, an open resource catalogue of human genetic variation [76]. The project is run by an international consortium and aims to describe over 90% of genetic variation down to 1% MAF. To date the genomes of more than 1000 individuals have been sequenced (with planned expansion to 2000 individuals in 2010) and around 10 million novel variants identified. The resource will enable the study of low frequency variants and aid fine mapping of regions of interest. Furthermore, next-generation deep sequencing technology is now available which will also increase the chances of rare variant detection [77]. All of this is carried out with the ambition to improve the diagnosis and treatment of this widespread condition.

## Figures and Tables

**Table 1:** Summary of recent GWAS of hypertension and/or blood pressure.

	Publication date	Phenotype	Discovery sample			Discovery and replication meta-analysis		
			*N*	OR/beta	Lowest *P*-value	*N*	OR/beta	*P*-value
Levy et al. [66]	Sept-07	DBP	1233	—	3.31 × 10^−6^	—	—	—
		SBP	1260	—	1.69 × 10^−6^	—	—	—
*Wang et al. [67]	Jan-09	SBP	542	—	7.6 × 10^−5^	7125	1.9	1.6 × 10^−7^
Org et al. [68]	Mar-09	hypertension	364/596	0.49	2.34 × 10^−6^	3808/4334	0.78	1.39 × 10^−6^
Cho et al. [69]	May-09	SBP	8842	−1.309	9.1 × 10^−7^	16703	−1.064	1.3 × 10^−7^
Adeyemo et al. [70]	Jul-09	DBP	8842	−0.882	1.2 × 10^−6^	16703	−0.63	3.0 × 10^−6^
		SBP	1017	—	4.72 × 10^−8^	—	—	—
		DBP	1017	—	.448	1997	—	.162
		hypertension	509/508	0.58	5.10 × 10^−7^	875/1122	—	.009

*Observed an SNP with a lower *P*-value but did not report on it as situated in gene desert.

—Value not reported (or in the case of meta-analysis replication not attempted).
